# Rib index, an objective measure to document changes of the rib hump deformity in a risser 4 progressive ais patient treated with the Schroth method

**DOI:** 10.1186/1748-7161-9-S1-O18

**Published:** 2014-12-04

**Authors:** Andrea Lebel, Victoria Lebel

**Affiliations:** 1 Ottawa and District Physiotherapy Clinic, Ottawa, Canada; 2 Saba University School of Medicine, Saba, Dutch Caribbean

## Background

The goals of Scoliosis Specific Schroth Physiotherapy (SSSPT) are to restore muscle balance, reshape the rib cage and reduce the rib hump to improve the esthetic of the patient, to increase the breathing areas and vital capacity, to reduce the Cobb angles, and to avoid spinal fusion surgery. Measuring the Cobb angles on P-A radiographs, assessing the sagittal profile on lateral radiographs, and calculating the Rib Index (RI), extracted from the Double Rib Contour Sign (DRCS) on lateral radiographs as one of the indicators of the rotational component of scoliosis, are three objective measurements Schroth physiotherapists can use to document the progression of this 3D condition. Traditionally, physicians have relied primarily on the Cobb angle when monitoring the progression and improvement of scoliosis. It is also important, however, to assess and document the rib cage rotation and rib hump size as reflected by the RI.

## Methods

A 13-year-old female was diagnosed with AIS in December 2011 and has been exclusively treated with SSSPT since September 2012. No bracing was used. The Cobb angle and RI from T6-T11 were measured on radiographs from December 2011 to October 2013. RI is the ratio of distances, d1/d2 (d1, the distance between the most extended point of the most extending rib contour to the posterior margin of the corresponding vertebra on lateral scoliosis film; d2, the distance from the least projected rib contour to the posterior margin of the same vertebra). The mean RI was calculated.

## Results

At diagnosis, the mean RI was 1.658 and the Cobb angle was 45°. In August 2012, the mean RI and

Cobb angle increased to 2.352 and 56°, respectively, indicating an increase in rib hump deformity and progression of scoliosis. In November 2012, after 12 weeks of SSSPT treatments, the mean RI and Cobb angle started to decrease to 2.16 and 52°. In October 2013, after a total of twelve 2-3hr clinic visits and 12 months of a daily 1.5-2hr Schroth HEP, the mean RI decreased to 1.665 and the Cobb angle to 42°. These mean RI and Cobb angle measurements show progression from 2011-2012 and improvement from 2012-2013 as a result of daily SSSPT.

## Conclusion

The RI can be used as an additional objective measure to show rib hump improvement with SSSPT.
